# Quantitative assessment of multichannel intraluminal impedance pH and its clinical implications

**DOI:** 10.14814/phy2.15199

**Published:** 2022-02-27

**Authors:** Eden Koo, John O. Clarke, Boli Yang, Pankaj J. Pasricha, Nina Zhang

**Affiliations:** ^1^ Division of Gastroenterology and Hepatology Michigan Medicine Ann Arbor Michigan USA; ^2^ John A. Burns School of Medicine University of Hawaii Honolulu Hawaii USA; ^3^ Division of Gastroenterology and Hepatology Johns Hopkins University School of Medicine Baltimore Maryland USA; ^4^ Division of Gastroenterology and Hepatology Stanford University School of Medicine Stanford California USA; ^5^ Department of Gastroenterology the Affiliated Drum Tower Hospital of Nanjing University Medical School Nanjing Jiangsu Province China; ^6^ Present address: Department of Digestive Diseases General Hospital of Jincheng Jincheng Shanxi China

**Keywords:** GERD, impedance, manometry

## Abstract

We sought to quantify the characteristics of acid reflux episodes in patients with extraesophageal GERD symptoms (EES), hiatal hernia (HH), and erosive esophagitis (EroE) using multichannel intraluminal impedance pH (MII‐pH) and investigate the correlation between impedance parameters and high resolution esophageal manometry (HREM). This was a retrospective analysis of esophageal manometric and impedance data inpatients with typical GERD symptoms who underwent both HREM and 24 h MII‐pH tests. Within the three patient subgroups, we evaluated impedance metrics such as average height of reflux, total duration of reflux, maximum duration of reflux, average pH, and average area of reflux. We also introduce a novel composite reflux index (CRI) metric, which is a measure of reflux height, duration, and acidity. Patients with EES exhibited a 29.3% increase in average height of reflux, compared to non‐EES patients (*p* < 0.01); the average height of reflux was found to be an independent predictor of EES (*p* < 0.01). Patients with HH showed a 190% longer total reflux duration (*p* < 0.01, vs. non‐HH patients). Total reflux duration was twice as long in EroE patients compared to those without (p = 0.02). Average CRI was significantly different within all three subgroup comparisons (*p* < 0.01). Impedance metrics shared weak negative correlations with lower esophageal sphincter (LES) rest pressure and distal contractile integral (DCI), and weak positive correlations with % absent peristalsis (*p* < 0.05 to *p* < 0.01 for various parameters). Quantitative impedance metrics provide useful insight into the pathophysiology of reflux in patients with EES, HH, and EroE.

## Graphical abstract

Quantitative impedance metrics such as average height of reflux and duration of reflux provides useful insight in the pathophysiology of reflux in patients with EES, HH, and EroE. According to impedance measurements, patients with EES demonstrate more proximal migration of reflux. Patients with HH and EroE have longer duration of reflux. The composite reflux index (CRI), a measure of reflux height, duration, and acidity may be a sensitive measurement of reflux severity.

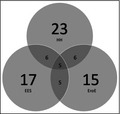

## 1 INTRODUCTION

Ambulatory reflux monitoring using multichannel intraluminal impedance pH (MII‐pH) technology is considered the “gold standard” for GERD diagnosis (Gyawali et al., 2018). In contrast to traditional pH monitoring, MII‐pH detects flow and directionality of intraluminal contents regardless of composition. MII‐pH allows for characterization of both acid and non‐acid reflux events as well as the proximal extent to which reflux has traveled in the esophagus (Nguyen et al., 1999; Sifrim et al., 1999).

In addition to typical GERD symptoms (heart burn, regurgitation), patients with GERD may also present, or even solely present, with “atypical” or extraesophageal symptoms (EES) that include asthma, laryngopharyngeal reflux, hoarseness, and chronic cough. Diagnosing this subset of patients is difficult, although MII‐pH workup has been proven to be useful (Cumpston et al., 2016; Sidhwa et al., 2017). One recent study demonstrated that 55–79% of patients with chronic hoarseness showed evidence of distal esophageal acid exposure (Vaezi et al., 2003). Other studies suggested that EES onset might be associated with non‐acid reflux, as detected by MII‐pH specifically (Vaezi et al., 2018).

Hiatal hernia (HH) is closely associated to GERD diagnosis (Duranceau, 2016). Studies have shown that the increased size of the HH is associated with more acid reflux (detected via ambulatory pH monitoring) and more severe forms of esophagitis (Jones et al., 2001; Kahrilas et al., 1999). However, the role of MII‐pH testing with regards to HH is still undefined and largely secondary to barium swallow/endoscopic evaluation.

Erosive esophagitis (EroE) describes a state of inflammation and esophageal damage secondary to esophageal reflux. Utilizing MII‐pH monitoring in a comparative study of patients with and without EroE, Savarino et al. was successful in showing that acid/non‐acid reflux episodes, volume, and acid clearance are important factors in the pathogenesis of esophageal mucosal damage (Savarino et al., 2010). Nonetheless, there is still very limited data comparing the impedance patterns of acid refluxes in patients with and without EroE.

Disorders of esophageal motility occur in a substantial amount of patients with GERD and contribute to increased esophageal exposure to refluxed acid material and reduced bolus clearance (Kahrilas et al., 1986). These include impaired peristalsis, hypotensive LES, and transient relaxation in contraction vigor (Gyawali et al., 2017; Tolone et al., 2017). There is evidence defining a proportional relationship between esophageal dysmotility and abnormal acid exposure (Kahrilas et al., 1986; Savarino et al., 2011). Nevertheless, little is known whether correlations exist between esophageal motility parameters assessed by high resolution esophageal manometry (HREM) and acid reflux events assessed by the MII‐pH.

Several parameters have been derived and utilized from the MII‐pH test, most commonly esophageal acid exposure time and symptom association analysis (Bredenoord et al., 2005; Wiener et al., 1988a, 1988b). More specific to MII‐pH testing, mean nocturnal baseline impedance and post reflux swallow‐induced peristaltic wave are parameters that have become increasingly adopted, but evidence on their utility is still emerging (Frazzoni et al., 2016). Previous groups have utilized impedance as a means to distinguish non‐acid and weakly acidic events (Bredenoord et al., 2008; Cumpston et al., 2016). To the best of our knowledge, the use of quantitative and automatic impedance analyses, such as reflux height, duration of reflux, area of reflux and composite reflux index assessed from the MII‐pH test have not previously been explored or implemented in patient studies. Although easily obtained from impedance tracings and data output, these parameters have been “overlooked” in favor of the current, widely adopted parameters. We believe that these “overlooked” measurements can provide useful physiological information that can further highlight the advantage of MII‐pH technology over traditional pH monitoring.

The aim of this retrospective study was to characterize reflux episodes in patients with EES, HH, and EroE using the aforementioned MII‐pH parameters assessed automatically by a special software, compare the difference in each of MII‐pH parameters among the three groups of patients, and investigate the correlation between the MII‐pH parameters and the HREM parameters.

## 2 METHODS

### 2.1 Subjects

From January 2011 to January 2015, patients referred to the Clinical Gastrointestinal Physiology Lab at Johns Hopkins Hospital were examined using the following inclusion criteria: (1) aged 18 years or older; (2) typical GERD symptoms occurring at least three times a week. Exclusion criteria included the following: (1) on acid suppression therapy before the esophageal manometric and impedance tests; (2) history of thoracic, esophageal, or gastrointestinal surgery; (3) malignant tumors of the esophagus or stomach; (4) systemic diseases affecting esophageal motility, such as diabetes mellitus or systemic sclerosis; (5) presence of severe organ dysfunction; (6) active psychiatric disease or disturbance of consciousness. All patients underwent esophagogastroduodenoscopy, HREM, and MII‐pH testing.

Patients were classified by the following for the purposes of three separate cohort analyses: (1) presence of extraesophageal symptoms (EES) that were judged to be clinically attributed to GERD, such as cough, wheezing, hoarseness, (2) hiatal hernia (HH) diagnosed previously via endoscopy or HREM, (3) erosive esophagitis (EroE) diagnosed previously via endoscopy. Because these phenotypes share an overlap (patients could have both EES and HH, for example) cohort assignment was not mutually exclusive.

### 2.2 High resolution esophageal manometry

A high‐resolution solid‐state manometry catheter (Medtronic, Los Angeles, CA) assembled with 36 circumferential sensors was intubated into the esophagus with the most distal sensor 3 cm within the stomach after 8 h or more of fasting (Ribolsi et al., 2020). After a brief period of acclimation with the catheter, the patient was instructed to swallow 5 ml of water, repeating for a total of 10 swallows. Derivation of metrics such as lower esophageal sphincter pressure (LES), distal contractile integral (DCI), and percentage of swallows with absent peristalsis was achieved by analysis of esophageal pressure topography (EPT). HREM/EPT data were analyzed using the ManoView software (Medtronic, Los Angeles, CA). Per the Chicago Classification (version 3.0), patients with major motility disorders including achalasia, distal esophageal spasm, and hypercontractile esophagus were excluded.

### 2.3 Multichannel intraluminal impedance pH

Upon the completion of the HREM test and removal of the HREM catheter, an MII‐pH catheter was inserted into the esophagus and connected with an external recorder. The esophageal impedance and pH were monitored on an ambulatory basis for a total of 24 h (Shay et al., 2004). The MII‐pH monitoring system (Medtronic, Duluth, GA) used in these patients was assembled with a catheter with one pH sensor positioned 5 cm above the LES (the LES location was determined by the HREM test) and six impedance sensors spaced at 3, 5, 7, 9, 15, and 17 cm proximal to the LES.

### 2.4 Assessment of impedance reflux parameters

The following MII‐pH parameters were derived and analyzed automatically via a custom‐written software that was validated with manual analysis: (1) Average height of reflux (cm) assessed as follows: first, the height of each reflux episode was determined from the impedance and then the heights of all reflux episodes within the entire 24‐h recording period were averaged for each subject; (2) maximum duration of acid reflux with pH < 4 (s): the episode with the longest duration among all reflux episodes; (3) total duration of all acid reflux episodes with pH < 4 (s) within the 24‐h period; (4) average acidity (pH) calculated as the average pH of all reflux episodes during the 24‐h period in each subject; (5) average area of all acid reflux episodes (cm s) calculated as follows: first, the area (reflux height × reflux duration) under the curve of each reflux period was calculated; then the areas of all reflux episodes were averaged for each subject; (6) average composite reflux index or CRI (cm s pH): CRI for each reflux episode was calculated as reflux height × reflux duration × acidity, and the average CRI is the average of CRIs of all reflux episodes in each patient. The numerical value for “pH” in this equation was the absolute value distance from a neutral pH of 7 (i.e., a reflux pH of 4 would be input into the CRI equation as “3”). Reflux height and durations were derived from impedance recordings.

As indicated above, the term “total” duration was defined as the sum duration of *all* recorded reflux episodes, averaged to account for each subject in a particular cohort. In contrast, the term “maximum” duration was defined as the *single* longest reflux episode recorded from each subject. To the best of our knowledge, the parameter of CRI had never been utilized or introduced in any previous studies. We believed this reproducible (via automated computation) metric would be a useful supplementary tool to provide objective, quantitative evidence of acid reflux severity.

### 2.5 Statistical analysis

Continuous variables are presented as mean ± standard deviation. For each cohort analysis, statistical comparisons were made with the unpaired two subject *t*‐test. Categorical variables are expressed as frequencies and percentages and compared via Chi‐Square analysis. Pearson's correlation coefficient (two‐tailed) was used to analyze the correlation between HREM and pH‐impedance parameters. To assess whether any of the impedance parameters might be considered risk factors for EES, HH, or EroE, multivariable logistic regression analyses were performed.

We identified potential confounding effects of mixed phenotypes (i.e., subjects having more than one phenotypes of EES, HH, and EroE) by comparing observed effect sizes in unadjusted and adjusted linear regression models. If the regression coefficient (ß) associated with a phenotype changes by >10% after adjusting for the additional two phenotypes, then we considered the phenotype to be a confounder.

*p* < 0.05 was considered statistically significant. All calculations were performed using SPSS (version 27, IBM Corporation, New York, USA).

### 2.6 Study approval

This retrospective study was prospectively reviewed and approved by the Institutional Review Board at Johns Hopkins Medicine.

## 3 RESULTS

### 3.1 Baseline demographics

**FIGURE 1:**
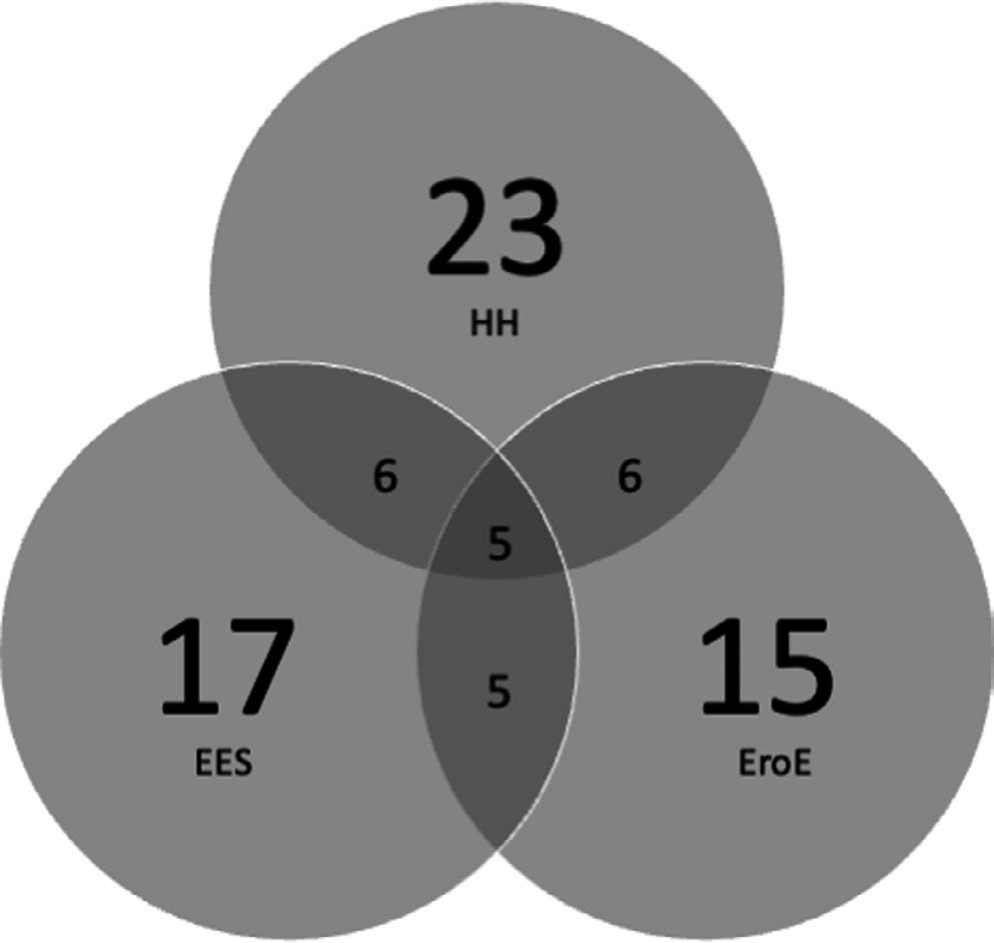
Venn diagram illustrating distribution of study participants among overlapping phenotypes

A total of 119 subjects meeting the criteria were included in the analysis (70 male, age: 25 to 73 years). The subjects were well balanced across all cohorts with respect to age and gender, as presented in Table 1. A total of 33 patients were identified to have EES, 40 patients had HH, and 31 patients had EroE. 4.2% (5/119) of patients had all three phenotypes, whereas 35.3% (42/119) had none of the phenotypes. Figure 1 describes overlapping phenotype group populations.

**TABLE 1:** Patient demographics

	Total number of subjects (*n* = 119)		
Mean age, y (SD)	49.6 (11.2)		
Female gender, *n* (%)	49 (41.2)		
	EES (*n* = 33)	Non‐EES (*n* = 86)	*p*‐value
Mean age, y (SD)	48.4 (10.6)	50.0 (11.5)	0.492
Female gender, *n* (%)	13 (39.4)	36 (41.9)	0.807
	HH (*n* = 40)	Non‐HH (*n* = 79)	*p*‐value
Mean age, y (SD)	50.9 (10.5)	48.9 (11.6)	0.351
Female gender, *n* (%)	18 (45.0)	31 (39.2)	0.546
	EroE (*n* = 31)	Non‐EroE (*n* = 88)	*p*‐value
Mean age, y (SD)	48.5 (9.6)	50.0 (11.8)	0.521
Female gender, *n* (%)	14 (45.2)	35 (39.8)	0.600

Note

*p*‐values obtained via chi‐square analysis.

Abbreviations: EES, extraesophageal symptoms; EroE, erosive esophagitis; HH, hiatal hernia; SD, standard deviation.

### 3.2 Comparison of impedance parameters in patients with and without EES

**FIGURE 2:**
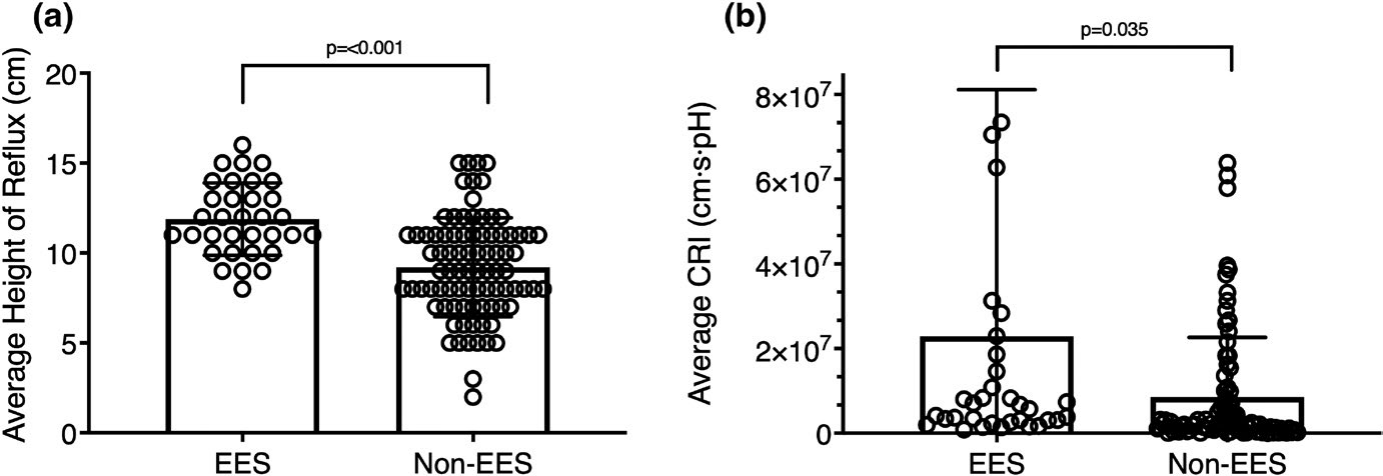
Differences between patients with (*n* = 33) and without (*n* = 86) extraesophageal symptoms when comparing impedance measurements of (a) average reflux height and (b) average composite reflux index (CRI). Data are presented as mean ± SD

According to impedance measurements, patients with EES experienced higher (i.e., more proximal) migration and longer episodes of acid reflux. Thirty‐three (27.7%) enrolled subjects reported EES, 11 of whom had underlying HH and 10 of whom had underlying EroE. The average height of reflux was 29.3% higher in the EES patients than the non‐EES patients (*p* < 0.01) (Figure 2). Both the maximum reflux duration (29.5 ± 44.3 s vs. 11.1 ± 16.8 s, *p* = 0.03) and the total reflux duration (119.7 ± 118.1 s vs. 63.6 ± 86.0 s, *p* < 0.01) were significantly longer in the EES group than the other patients. The CRI in the EES group was 2.67 times of that in the other patients (*p* = 0.03) (Figure 2). There was no difference in average pH (*p* = 0.11) or area of acid reflux between the patients with EES and without EES (*p* = 0.271).

### 3.3 Comparison of impedance parameters in patients with and without hiatal hernia

**FIGURE 3:**
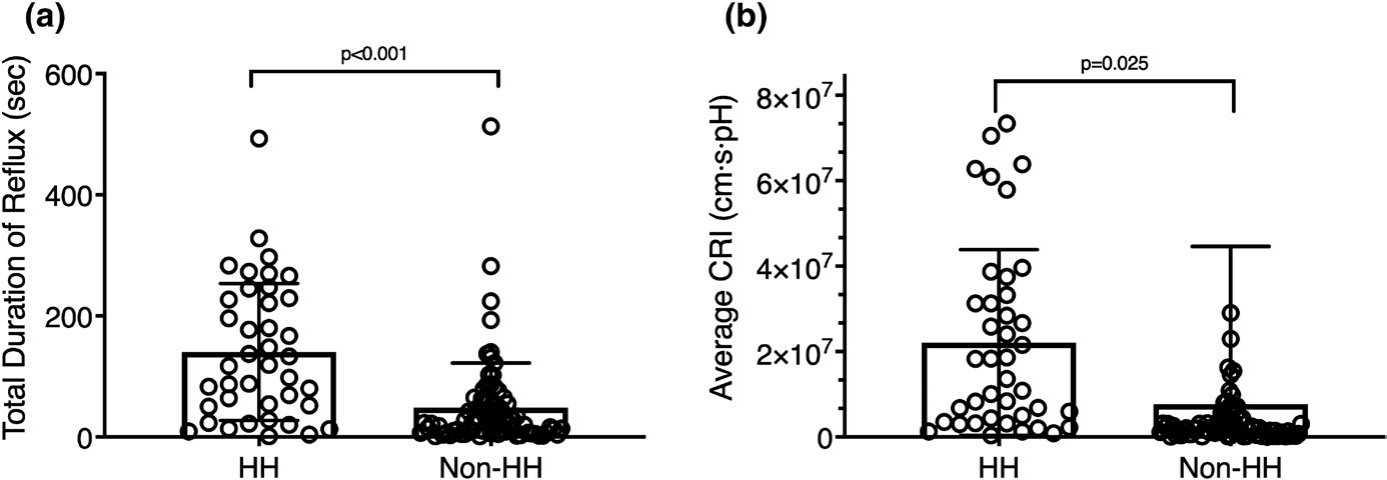
Differences between patients with (*n* = 40) and without hiatal hernia (*n* = 79) when comparing impedance measurements of (a) total duration of reflux (s) and (b) average composite reflux index (CRI). Data are presented as mean ± SD

Patients with HH showed significantly longer duration and greater area of acid reflux, according to the impedance measurements. Forty (33.6%) of enrolled subjects were diagnosed with HH, 11 of whom had EES and 11 of whom had EroE. These HH patients showed substantially longer maximum reflux duration (23.2 ± 22.5 s vs. 12.7 ± 30.4 s, *p* = 0.03, vs. other patients) and total reflux duration (140.3 ± 113.0 s vs. 48.3 ± 74.0 s, *p* < 0.01, vs. other patients) (Figure 3). The average area of reflux in the HH patients was three times of that in the other patients (3.0 × 10^5^ ± 3.4 × 10^5^ cm s vs. 1.0 × 10^5^ ± 5.0 × 10^5^ cm s, *p* = 0.03). This substantial difference was also reflected in the CRI (*p* = 0.03, HH vs. non‐HH) (Figure 3). There was no difference in the average pH (*p* = 0.104) or height of reflux between the two groups, although it is worth noting that the reflux height trended higher in the HH group (10.6 ± 2.5 cm vs. 9.6 ± 2.9 cm, *p* = 0.07, vs. other patients).

### 3.4 Comparison of impedance parameters in patients with and without evidence of EroE

**FIGURE 4:**
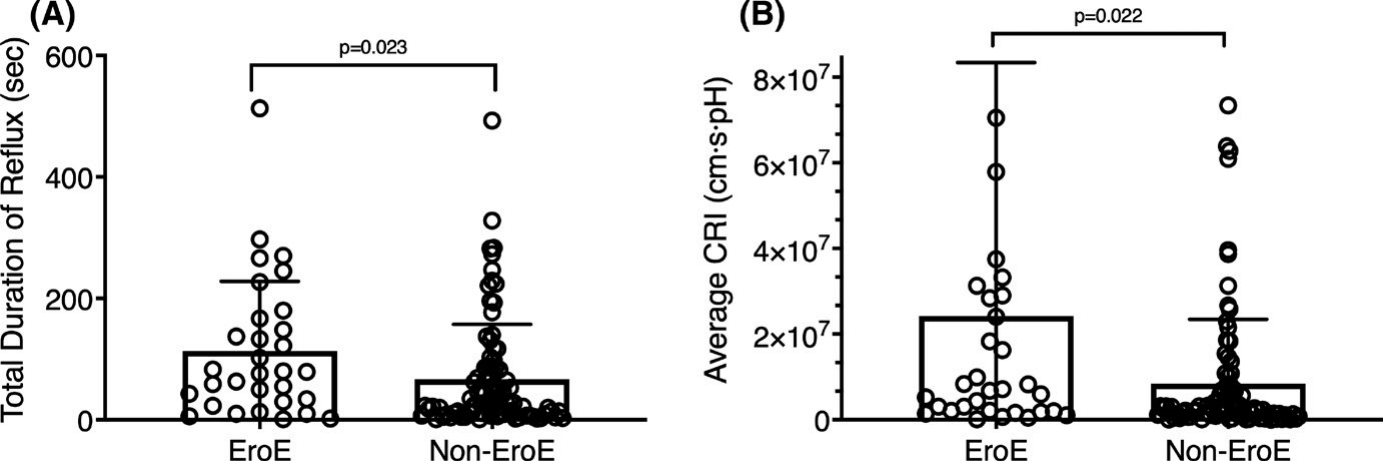
Differences between patients with (*n* = 31) and without (*n* = 88) erosive esophagitis when comparing impedance measurements of (a) total duration of reflux (s) and (b) average composite reflux index (CRI). Data are presented as mean ± SD

Thirty‐one (26.0%) subjects exhibited endoscopic evidence of erosive esophagitis, 10 of whom had EES and 11 of whom had HH. It was noted that the *total* duration of reflux (113.7 ± 114.5 s vs. 67.1 ± 90.2 s, *p* = 0.02) (Figure 4), rather than *maximum* duration (*p* = 0.152), was longer in the EroE cohort than that in the other cohort. The average area of reflux in the EroE patients was 3.64 times of that in the other patients (3.6 × 10^5^ ± 8.2 × 10^5^ cm s vs. 9.9 × 10^4^ ± 2.0 × 10^5^ cm s, *p* < 0.01). The difference in the CRI was also substantial: the CRI in the EroE patients was 2.86 times of that in the non‐EroE patients (*p* = 0.02) (Figure 4). There was no difference in average reflux height (*p* = 0.32) or average pH (*p* = ns).

### 3.5 Impedance parameters as independent risk factors of EES, HH, and EroE

We performed a multivariable logistic regression analysis to assess if MII‐pH parameters could independently predict, and hence be considered “risk factors” factors for, EES (Table 2), HH, and EroE. Larger average height of reflux predicted an increased likelihood of having EES (OR = 1.59, *p* < 0.01). Longer total duration of reflux was associated with a slight increased likelihood of having HH (OR = 1.02, *p* < 0.01). None of the parameters were independently predictive of EroE.

**TABLE 2:** Logistic regression analysis of risk factors for EES

Reflux parameter	Unadjusted OR (95% CI)	*p*‐value	Adjusted OR (95% CI)	*p*‐value
Average height of reflux	1.530 (1.256–1.863)	<0.001	1.592 (1.270–1.996)	<0.001
Maximum duration of reflux	1.026 (1.006–1.046)	0.012	1.041 (0.992–1.093)	0.105
Total duration of reflux	1.005 (1.001–1.010)	0.010	1.000 (0.992–1.008)	0.937
Average pH	0.682 (0.427–1.091)	0.110	0.825 (0.459–1.482)	0.519
Average area of reflux	1.000 (1.000–1.000)	0.192	1.000 (1.000–1.000)	0.066
Average CRI	1.000 (1.000–1.000)	0.120	1.000 (1.000–1.000)	0.217

Note

*p* values obtained via binary logistic regression analysis.

Abbreviations: CI, confidence interval; OR, odds ratio.

### 3.6 Identifying confounding effects of phenotypes on impedance measurements

HH and EroE did not have confounding effects on the association between EES and average reflux height (unadjusted ß = 2.67, adjusted ß = 2.65; *p* < 0.01 for both models). Similarly, EES and EroE were not confounders on the association between HH and total reflux duration (unadjusted ß = 92.05, adjusted ß = 91.34; *p* < 0.01 for both models). Because the regression coefficient for the model describing the relationship between EroE and total reflux duration decreased by >10% (unadjusted ß = 46.63, adjusted ß = 41.02; *p* < 0.01 for both models), the presence of underlying EES and HH might be considered confounders in this relationship.

### 3.7 Correlations between impedance parameters and esophageal motility parameters

**FIGURE 5:**
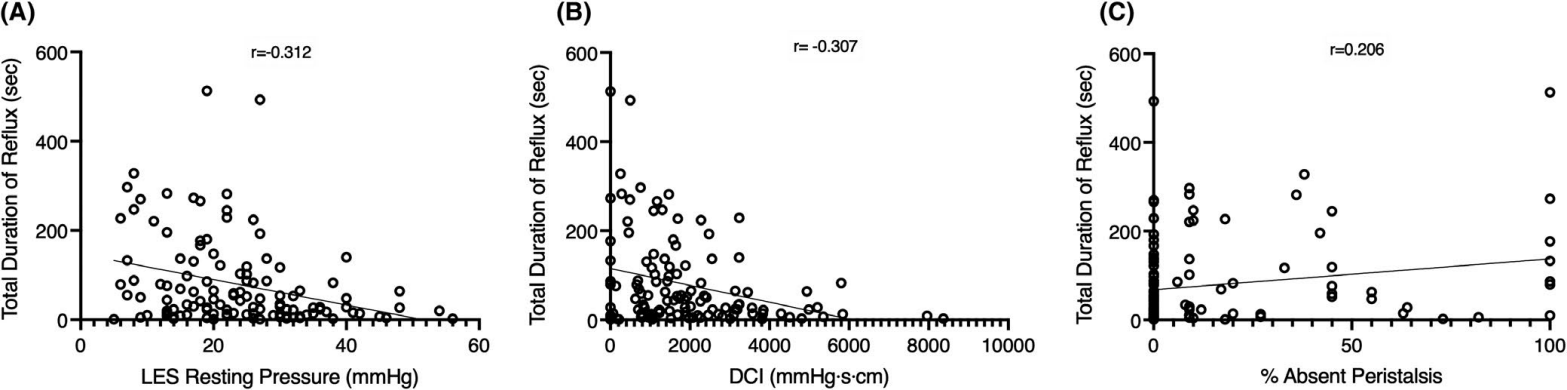
Shown above are scatter plots illustrating the relationship of total reflux duration compared with esophageal motility parameters (*n* = 119, complete data set). The fitted lines reflect a simple linear regression analysis with corresponding Pearson correlation coefficients

The impedance measurements for maximum duration, total duration, and average area of reflux were found to be weakly correlated with the HREM parameters. Findings from the analysis for correlations between impedance and HREM parameters are summarized in Table 3. The total duration of reflux, specifically, shared the strongest (albeit weak) inverse correlation with LES rest pressure (r = −0.312, *p* < 0.01) and DCI (r = −0.307, *p* < 0.01). A scatterplot summary of these results is shown in Figure 5. Although no causal relationship can be inferred, this can be interpreted that longer durations of acid reflux events are related to weak/absent vigor of esophageal contractions.

**TABLE 3:** Correlations of impedance measurements and esophageal motility parameters

	LES resting pressure(mmHg)		DCI (mmHg s cm)		% absent peristalsis	
	Correlation coefficient	*p‐value*	Correlation coefficient	*p‐value*	Correlation coefficient	*p‐value*
Average height of reflux	−0.188*	0.041	−0.161	0.080	0.107	0.246
Maximum duration of reflux	−0.193*	0.036	−0.201*	0.029	0.236*	0.010
Total duration of reflux	−0.312**	<0.001	−0.307**	<0.001	0.206*	0.024
Average acidity	0.047	0.610	0.167	0.070	−0.018	0.846
Average area of reflux	−0.192*	0.037	−0.203*	0.027	0.289**	0.001
Average composite reflux index	−0.190*	0.039	−0.211*	0.021	0.296**	0.001

Note

*p < 0.05 and **p < 0.01, using Pearson's correlation analysis.

Abbreviations: DCI, distal contractile integral; LES, lower esophageal sphincter.

## 4 DISCUSSION

We compared the reflux parameters derived from the MII‐pH test between patients with and without EES, HH, or EroE. Our findings showed more proximal migration, longer duration of acid refluxate episodes, and larger CRI in patients with EES compared to non‐EES. Compared to their opposites, patients with HH exhibited longer duration of reflux episodes, in addition to larger reflux “area” (a product of reflux height and duration) and larger CRI. As for patients with EroE in comparison with those without EroE patients, they similarly showed longer *total* reflux duration, larger reflux area, and CRI. The novel parameter, CRI distinguishes between patients within the studied phenotypes (with or without EES, HH, or EroE). Finally, we found that the impedance measurements shared weak correlations with some esophageal manometry parameters.

The observed difference in reflux height in patients with EES vs. patients with non‐EES might be attributed to either a “stronger” reflux or a larger volume of reflux that forcibly travels higher up the esophagus. The pattern is of particular interest to us as it supports established mechanisms in the pathogenesis of EES. For example, chronic cough due to reflux was thought to be caused by gastric reflux directly irritating the tracheobronchial tree after aspiration into the airway. This triggers either the afferent limb of the cough reflex or an esophageal‐bronchial neural cough reflex (Irwin, 2006). Laryngopharyngeal reflux similarly could result from gastric reflux traveling beyond the upper esophageal sphincter and onto the pharyngeal tissue (Ford, 2005). In some cases, asthma was reported to result from bronchoconstriction that was induced by reflux irritating the tracheobronchial tree or activating a vagal neural reflex arc in the esophagus (National Asthma Education & Prevention Program, 2007). With regards to the difference in both total and maximum duration of acid reflux episodes shown in EES vs. non‐EES patients, one possible explanation could be that there were primarily more frequent reflux episodes in these patients. However, the duration of reflux might also be secondary to the higher proximal reflux migration, as these episodes would naturally take longer to clear. Another secondary explanation could be the prevalence of underlying HH amongst our EES subjects, as this factor has been proven to promote acid reflux. We also suspect that any additional mechanism that reduces the clearing capacity of the esophagus, such as compromised esophageal peristalsis, may contribute towards longer reflux durations. In corroboration with our findings, Ribolsi et al. also reported the role of either longer reflux duration or more frequent reflux in the initiation of EES (Ribolsi et al., 2014). The cause of the increased reflux height was not studied in the present study and deserves further investigation. It could result from the increased pressure gradient between the stomach and the esophagus, such as increased gastric pressure. The weak but significant negative correlation between the LES pressure and the reflux heighted suggested a role of the reduced LES pressure.

The presence of HH, particularly Type 1 sliding hernia, is strongly associated with symptomatic GERD. Previous studies suggested that 50–94% of patients with GERD had a Type 1 HH, as opposed to 13–59% of control subjects (Ott et al., 1985; Wright & Hurwitz, 1979). Detailed physiological experiments have concluded that susceptibility to GERD is proportional to the size of hernia. Proposed mechanisms describe hernias compromising the ability of the gastroesophageal (GE) junction to prevent reflux, as well as inhibiting esophageal acid clearance (Buttar & Wang, 2004; Chait, 2010). Therefore, it makes sense to us that the impedance measurement detected the prolongation in acid clearance via longer total and maximum reflux durations in HH subjects. Given that the reflux area is a product of reflux duration and height of reflux, it is unsurprising that this parameter was significantly larger in the HH vs. non‐HH patients. Because there was no significant difference in height of reflux between the two groups, the absolute difference in reflux duration between the two groups is further emphasized. This example also unveils a potential limitation of the reflux area parameter, as analyzing this alone would give investigators unclear conclusions as to which underlying factor(s) truly contribute to statistical comparisons.

As for the EroE compared to non‐EroE subjects, it was interesting to note that the *total* duration, rather than the *maximum* duration, was significantly higher. These findings suggested that repeated and more frequent insults, rather than a single more “intense” maximum insult, might be responsible for esophageal mucosal injury seen in EroE. It can be hypothesized that duration of reflux events are dependent on a lag in the initiation of “secondary peristalsis”, as well as a defect in reflux perception (Barham et al., 1992, 1995; Chen et al., 2014). Savarino et al. has previously shown that MII‐pH monitoring could be clinically relevant in differentiating erosive and nonerosive GERD; specifically, the study concluded that the number of reflux episodes played an important role in the pathogenesis of esophageal mucosal damage (Savarino et al., 2010). This corroborates our data regarding longer total duration in patients with EroE. It should be noted, however, that this relationship may not be as strong due to confounding effects of subjects with underlying HH and/or EroE. However, the previous study also detected a higher percentage of reflux episodes reaching the proximal esophagus in EroE subjects compared to non‐EroE. Whereas, our study revealed the lack of difference in reflux height between patients with and without EroE.

The novel parameter, CRI was significantly greater in all three GERD phenotypes when compared to their opposites. The absolute differences in CRI amongst the three comparisons were quite large, specifically on an entire order of magnitude. These findings suggest that when taken into context with other diagnostic parameters (for example baseline impedance, acid exposure time), the CRI metric may be a useful supplementary tool to provide evidence of acid reflux severity. Though highly speculative, it is exciting to envision to possibility of utilizing a simple, reproducible metric to further quantify an aggregate of acid severity, duration, and proximal height. Further clinical studies are warranted to explore clinical implications of this novel parameters in various groups of patients with GERD, specifically if the CRI can be defined in both GERD and non‐GERD patients and validate an association with a risk of additional phenotype (such as EES or EroE).

The weak but significant correlations between the selected impedance and HREM parameters is further evidence of the cause‐effect interplay between esophageal motility disorders and esophageal reflux. Both LES resting pressure and DCI share inverse relationships with both reflux duration and height. Amongst previously well‐described mechanisms, the relaxation of the LES allows gastric reflux to travel proximal towards the esophagus, especially when patients are in supine positions (Liu et al., 2019). Both weak vigor and absence of peristalsis inhibits the ability to “suppress” bolus contents from traveling proximally, thus explaining the positive, significant correlations between % absent peristalsis and the impedance measurements.

There were several limitations in this study. Most importantly, no conclusions regarding the diagnostic or therapeutic implications of the MII‐pH parameters could be derived from this study. The aim of our analysis was not to compare the clinical utility of the analyzed impedance parameters against previously established ones, such as acid exposure time, symptom association analysis, baseline impedance, peristaltic wave, or manometric parameters. We recognize that our findings currently cannot yet support a use of the CRI as a practical tool for clinicians to incorporate in GERD diagnostics (or even management trees), but it is our hope that the introduction of this metric will pave way for this in the future. Our retrospective study should be viewed as hypothesis generating in this regard. Further, we limited our characterization of HH and EroE phenotypes, choosing not to specifically categorize by hernia type (sliding, paraesophageal) or by esophagitis grade (per LA Classification). This was done to simplify the analysis patterns for the purpose of general reproducibility and further clinical studies should be done to determine the effect of phenotype classification on impedance measurements (e.g., the effect of hiatal hernia size on CRI).

In conclusion, the quantitative parameters derived from the MII‐pH test are capable of differentiating *within* a number of subgroups of patients with GERD. We find that when using impedance measurements, higher reflux height is suggestive of extraesophageal symptoms and a longer reflux duration is indicative of hiatal hernia and implicative of erosive esophagitis. The CRI is an automated, easily reproducible novel metric that can also differentiate within GERD phenotypes while as a general marker of reflux severity.

## CONFLICT OF INTEREST

None.

## AUTHOR CONTRIBUTION

EK, NZ, and BY analyzed the data and drafted the manuscript; John_C and PP interpreted MII and HREM data; all authors contributed to the preparation of the manuscript.

## 5 ETHICAL APPROVAL

Informed, written consent was obtained from all study participants. This retrospective study was prospectively reviewed and approved by the Institutional Review Board at Johns Hopkins medicine.
